# Clinical features and prognosis of pregnancy-related renal damage and pregnancy after chronic kidney disease

**DOI:** 10.1186/s12884-023-05941-7

**Published:** 2023-08-29

**Authors:** Li Fang, Bingbing Shen, Huhai Zhang, Na Yin, Juan Cai, Jun Zhang, Hongwen Zhao

**Affiliations:** 1 Department of Nephrology, First affiliated hospital, Third Military Medical University (Army Medical University), Chongqing, 400038 China; 2 Department of Nephrology, Chongqing University Central Hospital, Chongqing Emergency Medical Center, NO.1 Jiankang Street,Yuzhong District, Chongqing, 400014 China

**Keywords:** Pregnancy, Renal damage, Chronic kidney disease, Renal biopsy, Pathology

## Abstract

**Objective:**

To explore the clinical features of renal damage related to pregnancy and pregnancy after chronic kidney disease (CKD), providing clinical evidence for the relationship between renal damage and pregnancy.

**Methods:**

A retrospective analysis was performed on patients admitted to our hospital between March 2013 and February 2021 who had both pregnancy and kidney damage. The study collected pathology results from renal biopsies, 24-hour urinary protein quantity, albumin (Alb), serum creatinine (Scr), blood lipids, coagulation function, blood routine, and other indicators during and after pregnancy.

**Results:**

This study included 82 cases, with 48 cases in the pregnancy-related renal damage group. Thirty-four cases were in the post-CKD pregnancy group. Of the patients, 30 cases (88.24%) had CKD stage 1–2. Results showed better pregnancy and fetal outcomes in the post-CKD pregnancy group compared to the pregnancy-related renal damage group (Ρ was 0.029 and 0.036, respectively). Renal biopsy pathology revealed that 16 cases (33.33%) in the pregnancy-related renal damage group mainly had focal segmental glomerulosclerosis (FSGS), while the post-CKD pregnancy group was dominated by 14 cases (43.75%) of IgA nephropathy. The first blood test indicators revealed that the pregnancy-related renal damage group had lower estimated glomerular filtration (eGFR) and Alb levels compared to the post-CKD pregnancy group (Ρ was 0.003 and 0.000, respectively). Additionally, 24-hour urinary protein quantity, total cholesterol (Tch), triglyceride (TG), and platelet (PLT) counts were higher in the pregnancy-related renal damage group compared to the post-CKD pregnancy group (Ρ was 0.005, 0.001, 0.008, and 0.031, respectively). The abnormal rate of Scr during pregnancy was 41.67% (20/48) in the pregnancy-related renal damage group and 17.39% (4/23) in the post-CKD pregnancy group, with a statistically significant difference (Ρ was 0.043).

**Conclusion:**

The pregnancy-related renal damage group is mainly associated with FSGS, while the post-CKD pregnancy group is characterized by IgA nephropathy. Patients with CKD1-2 can have a successful pregnancy after achieving good control of eGFR, albumin, 24-hour urinary protein quantity and other indicators, resulting in better pregnancy and fetal outcomes. Abnormal Scr levels during pregnancy of pregnancy-related renal damage can be improved within 3 months after delivery.

## Introduction

Proteinuria, hematuria, edema and abnormal renal function are common clinical manifestations of renal injury [1]. Infections and drugs are significant factors resulted in kidney damage [2, 3]. Pregnancy may also play a vital role in this regard [4]. Pregnancy-related acute kidney injury is a common and serious pregnancy complication [5, 6]. Chronic kidney disease (CKD) is a global health problem [7]. A literature report [8] that although the prevalence of CKD in women of childbearing age seems relatively low, with estimates on the order of 0.1–4%, the implications of pregnancy in the context of CKD are many and can be severe. Pregnancy outcomes in renal disease depend on baseline creatinine levels, the degree of proteinuria, and hypertension, and there is an increased risk of maternal-foetal complications and CKD progression with worsening renal function [9]. Currently, clinicians lack adequate understanding of pregnancy-related kidney damage. Consequently, there is a scarcity of research on clinical characteristics and prognosis of pregnancy-related kidney damage and post-CKD pregnancy. This study aims to compare the differences in renal pathology characteristics, pregnancy outcomes, and biochemical indicators between pregnancy-related kidney damage and post-CKD pregnancy. The findings of this study will help deepen awareness of the disease and provide a basis for the diagnosis and treatment of kidney damage during pregnancy.

## Methods

A retrospective analysis was conducted on the clinical data, delivery information, and follow-up of cases with pregnancy-related nephropathy in our hospital from March 2013 to February 2021. The cases were split into two groups based on whether there had been pre-existing renal damage: the group with pregnancy-related renal damage and the group with post-CKD pregnancy. Prior to pregnancy, both groups had no history of pregnancy, underlying diseases, and positive results on urinalysis (urine protein and occult blood) and renal function tests. This study received approval from the hospital ethics committee (Approval number: KY2019143).

### Inclusion criteria

Group with pregnancy-related renal damage: No evidence of renal damage is detected prior to pregnancy through clinical signs and laboratory examinations. However, during pregnancy, there is presence of proteinuria (nondaily cleaning of the middle urine protein qualitative + and above or 24 h urine protein quantitative ≥ 0.3 g). Clinical manifestations of renal damage may or may not be present, such as edema, hypertension, etc. Renal damage is confirmed through complete laboratory examinations and pathological results of kidney biopsy taken during pregnancy or postpartum.

Group with post-CKD pregnancy: Prior to pregnancy, there is a clear history of chronic kidney disease (CKD) with abnormal renal structure or function affecting health for more than 3 month [1, 10]. The medical history, laboratory tests and pathological results of renal biopsy are complete.

### Exclusion criteria

The medical history, laboratory tests and pathological results of renal biopsy are incomplete.

The study gathered and analyzed data pertaining to age, gestational age, pregnancy termination time, delivery method, preterm birth, weight, renal puncture time, pathological type, and laboratory indicators. Pathological diagnosis, classification methods, and CKD staging were based on the KDIGO 2021 clinical practice guideline for the management of glomerular diseases [1, 11].

### Definitions

Scr > 77 µmol/l (0.87 mg/dl) was considered the upper limit for pregnancy [9]. Preterm birth refers to births occurring between 28 and 37 weeks of gestation. Low birth weight infants have a birth weight of less than 2500 g, while very low birth weight infants weigh less than 1500 g. Miscarriage describes the spontaneous termination of pregnancy within the first trimester, generally within 12 weeks. Induction of labor primarily refers to terminating pregnancy beyond 12 weeks of gestation.

### Statistics

SPSS 18.0 statistical software(IBM,Chicago,USA) was used for statistical analysis, and measurement data are expressed as mean ± standard deviation (${\bar x }$±SD)and were analysed by t test. Data that did not meet the normal distribution are represented as the medians (M). The nonparametric method was used for uneven variance and the rank-sum test (Mann-Whitney test). The counting data were statistically analysed using the chi-square test. P < 0.05 was considered statistically significant.

## Results

### General information

A total of 82 patients were involved in the study. The pregnancy-related renal damage group included 48 patients who met the inclusion criteria. Among them, 20 cases were diagnosed with acute kidney injury and 21 cases were diagnosed with preeclampsia. Of the 21 cases, 12 were diagnosed with acute kidney injury, while 18 were diagnosed with nephrotic syndrome of pregnancy (NSP). The average age of gestation was 27.21 ± 4.33 years, with a range of 20–37 years, and the gestational age varied from 8 to 40 + 6 weeks, with an average of 31.94 + 2.13 ± 8.39 + 1.83weeks. Out of the 48 cases, 11(22.92%) experienced pregnancy termination, out of which 7 were induced, and 4 were miscarriages.

In the post-CKD pregnancy group, a total of 34 patients who met the inclusion criteria were enrolled in the study. Of these cases, 10 were diagnosed with severe preeclampsia, 4 with chronic renal insufficiency, and 5 with gestational diabetes mellitus type A1. CKD staging was determined based on pre-pregnancy eGFR, with 26 cases (76.47%) classified as CKD stage 1, 4 as CKD stage 2, 3 as CKD stage 3, and 1 as CKD stage 5. The average age of gestation was 29.44 ± 4.05 years, with a range of 23–39 years, and the gestational age ranged from 30^+ 1^ to 40^+ 1^ weeks. Pregnancy was terminated in only one case (2.94%) due to induced labor. The post-CKD pregnancy group had better pregnancy and fetal outcomes compared to the pregnancy-related renal damage group (P values of 0.029 and 0.036, respectively), while there was no significant difference in fetal weight between the two groups (P = 0.059, > 0.05). Detailed data are presented in Tables 1, 2 and 3.

**Table 1:** Comparison of pregnancy outcomes

Group	Pregnancy-related renal damage(N = 48)	Post-CKD pregnancy(N = 34)
Natural labour	6	8
Caesarean section delivery	31	25
Termination of pregnancy	11	1

*Ρ-value* 0.029

**Table 2:** Comparison of foetal outcomes

Group	Pregnancy-related renal damage(N = 48)	Post-CKD pregnancy(N = 34)
Term delivery	19	19
Premature birth	18	14
Stillborn	11	1

*Ρ-value* 0.036

**Table 3:** Comparison of foetal weight

Group	Pregnancy-related renal damage(N = 37)	Post-CKD pregnancy (N = 33)
Low weight	15	5
Very low body weight	4	4
Normal weight	18	24

*Ρ-value* >0.05

### Characteristics of renal biopsy

All 48 patients in the group with renal damage related to pregnancy underwent renal biopsy. The renal biopsies were performed between 5 and 1110 days postpartum, with a median of 66 days. Four patients underwent biopsy one year after giving birth. Of these biopsies, 16 cases (33.33%) showed FSGS, with one case being complicated by acute tubular necrosis. 12 cases (24.99%) displayed mesangial proliferative glomerulonephritis, of which two were complicated with acute tubular injury, and one was complicated with interstitial chronic inflammation. IgA nephropathy was diagnosed in 10 cases (20.84%), and lupus nephritis was found in 8 cases (16.68%).

In the post-CKD pregnancy group, 32 patients received renal biopsy before pregnancy. However, one patient with uremia and another one with right renal atrophy did not undergo renal biopsy. Of the biopsies performed, 14 cases (43.75%) showed IgA nephropathy, including two cases of purpuric nephritis. Lupus nephritis was diagnosed in 12 cases (37.5%), as reported in Table 4.

**Table 4:** Characteristics of the distribution of renal pathological types

Pathological type	Pregnancy-related renal damage group(N = 48) (case/ percentage)	Post-CKD pregnancy group(N = 32) (case/ percentage)
FSGS	15(31.25%)	1(3.12%)
IgA nephropathy	10(20.84%)	12(37.5%)
Mesangial proliferative glomerulonephritis	9(18.75%)	5(15.63%)
Lupus nephritis	8(16.68%)	12(37.5%)
Diabetic nephropathy with interstitial severe inflammatory lesions	1(2.08%)	0
Kidney damage to undifferentiated connective tissue	1(2.08%)	0
Focal segment glomerulosclerosis with acute tubular necrosis	1(2.08%)	0
Mesangial proliferative glomerulonephritis with acute tubular necrosis	1(2.08%)	0
Mesangial proliferative glomerulonephritis with chronic interstitial inflammation	1(2.08%)	0
Mesangial proliferative glomerulonephritis with acute tubular interstitial kidney injury	1(2.08%)	0
Purpura nephritis		2(6.25%)

### Comparison of the first blood test indicators during pregnancy among two groups: pregnancy-related renal damage and post-CKD pregnancy groups

The first indicators during pregnancy had no time limit according to real clinical data. In comparison to the post-CKD pregnancy group, the pregnancy-related renal damage group exhibited lower eGFR and Alb levels (P = 0.003 and 0.000, respectively). However, the pregnancy-related damage group had higher levels of 24-hour urinary protein quantity, Tch, TG, and PLT in comparison to the post-CKD pregnancy group (P = 0.005, 0.001, 0.008, and 0.031, respectively), as presented in Table 5.

**Table 5:** Comparison of the first blood test indicators during pregnancy between the two groups

blood test indicators	Pregnancy-related renal damage group(N = 48)	Post-CKD pregnancy group(N = 34)	*Ρ-value*
24-h urine protein(mg/24 h)	1996.00	1137.32	0.005
eGFR(ml/min/1.73m^2^)	93.50 ± 38.49	121.70 ± 44.80	0.003
CR(umol/L)	91.08 ± 69.03	75.50 ± 98.09	>0.05
Alb(g/L)	32.19 ± 7.22	37.72 ± 4.78	0.000
Tch(mmol/L)	6.52	5.06	0.001
TG(mmol/L)	3.57	2.74	0.008
LDL-C(mmol/L)	3.75 ± 1.66	3.57 ± 0.98	>0.05
WBC(10^9^/L)	8.16 ± 2.47	10.19 ± 12.69	>0.05
Hb(g/L)	116.91 ± 13.32	117.41 ± 16.86	>0.05
PLT(10^9^/L)	257.96	216.26	0.031
D-Di(mg/L)	1.76 ± 2.97	4.29 ± 13.05	>0.05
Fib(g/L)	5.20	4.82	>0.05

Data are expressed as mean ± standard deviation (${\bar x }$±SD)、 medians (M)

### Renal prognosis

In this study, a total of 71 cases were followed up for more than 3 months after delivery, with each case having a follow-up time of over 1 year. The abnormal Scr levels during pregnancy were identified as the first Scr > 77 µmol/L, while postpartum Scr abnormality was represented by the first Scr > 84 µmol/L at a postpartum follow-up of over 3 months. In the pregnancy-related renal damage group, 41.67% (20/48) of cases exhibited abnormal Scr during pregnancy while this was only 17.39% (4/23) in the post-CKD pregnancy group. Similarly, the rates of Scr abnormalities during pregnancy were significantly higher in the pregnancy-related renal damage group (P = 0.043), as shown in Table 6. Postpartum Scr abnormalities were observed in 29.17% (14/48) of cases in the pregnancy-related renal damage group and 8.7% (2/23) in the post-CKD pregnancy group. However, there was no statistically significant difference between the two groups regarding postpartum Scr abnormalities. There was no statistically significant comparison of pregnancy and postpartum Scr abnormalities in each group.

**Table 6:** Comparison of abnormal rates of creatinine during pregnancy

Group	Pregnancy-related renal damage(N = 48)	Post-CKD pregnancy (N = 23)
Normal creatinine	28	19
Abnormal creatinine	20	4

*Ρ-value* 0.043

## Discussion

Research indicate [12] that in pregnant women who have preexisting kidney disease, approximately 43% may experience pregnancy-related dysfunction after pregnancy, and 10% will experience rapid deterioration of renal function. What renal function and renal pathology changes that occur during pregnancy in women without preexisting renal damage, and how are they different from those in pregnant women after CKD? At present, there are few reports, so this study mainly explores the clinical features of both groups.

Koratala et al. [13] discover a correlation between renal impairment during pregnancy and adverse pregnancy outcomes. The study revealed that in the pregnancy-related kidney damage group, the fetal survival rate was 77.1%, consistent with earlier reports [14]. In the post-CKD pregnancy group, 25 patients (73.5%) underwent caesarean section, and the fetal survival rate was 97.06%, which was similar to that reported in the literature [15]. We concluded that the pregnancy outcomes in the post-CKD pregnancy group were better than in the pregnancy-related renal damage group. The termination of pregnancy rate was higher in the latter group, with a 22.92% (11/48) increase, and detectably, out of 11 patients who underwent postpartum renal biopsy, 7 showed FSGS, with six of them having clinical manifestations of NSP. These findings suggest that the probability of pregnancy termination when NSP occurs during pregnancy is high, with FSGS being the primary pathological manifestation of the kidneys. Fetal outcomes were better in the post-CKD pregnancy group. Overall, patients with stages 1–2 of CKD had more favorable pregnancy and fetal outcomes compared to patients with pregnancy-related kidney damage.

Renal biopsy is the main diagnostic basis for determining the cause of kidney damage, and it is not recommended to perform renal biopsy for patients who are pregnant for more than 30 weeks because of the increased risk of preterm delivery and fetal death [16]. Preeclampsia affect the endothelial cells and podocytes of the glomerulus, which may easily lead to NSP. In the pregnancy-related renal damage group, eighteen cases of NSPs were found, and cellular changes disappeared after delivery. Proteinuria typically ceased by 12 weeks postpartum. But some research [17, 18] have found that podocytes persist postpartum, leading to FSGS. Our research showed that renal biopsies were performed in the postpartum period for the pregnancy-related renal damage group. Due to the high amount of proteinuria and elevated Scr during pregnancy or postpartum, approximately 50% of patients underwent a kidney biopsy within 3 months after delivery. FSGS were the main pathological types. FSGS accounted for 7 cases in NSP. 10 cases of IgA nephropathy were confirmed by postpartum renal biopsy, and the possibility of nephropathy during pregnancy was high. Lupus nephritis may be considered to be related to the hormone secretion during pregnancy and the lack of prenatal immune system examination. There were 3 cases of acute tubular necrosis, which may be due to the hypercoagulable state of NSP. In the post-CKD pregnancy group, the main pathological types were consistent with the KDIGO 2021 Clinical Practice Guideline [1].

According to the literature, [19] many pregnant women with CKD can choose to stop or reverse the progression of kidney disease after terminating their pregnancy, especially if kidney disease is worsening. We found that some blood test indicators were well controlled for the post-CKD pregnancy group. However, the indicators of pregnancy-related renal damage group was poor, likely due to renal damage was first detected during pregnancy without early prevention intervention. Consequently, the pregnancy is recommended with caution and termination it if necessary to prevent adverse pregnancy outcomes. After the above indicators of CKD are controlled, active pregnancy can be performed, especially in patients with CKD stage 1–2.

This study found that the rate of Scr abnormalities during pregnancy in the two groups was statistically significant (P < 0.05), and the other comparisons were meaningless. It was found that women with pregnancy-related renal damage were more likely to experience renal function abnormalities during pregnancy, which improved three months after giving birth. These findings suggest the importance of early comprehensive examination and intervention during pre-pregnancy and early pregnancy. However, literature reports indicate [20] that preeclampsia(PE) is associated with kidney dysfunction due to deficiencies in podocyte-specific vascular endothelial growth factor (VEGF). Glomerular endotheliosis and thrombotic microangiopathy (TMA) are the main features of kidney involvement in PE. This complex pathophysiology is ameliorated after delivery; however, permanent kidney damage may remain and is intensified thereafter.

In addition, in the pregnancy-related renal damage group, only one case displayed albumin levels equal to or greater than 30 g/L and a 24-hour urine protein quantification exceeding 3500 mg. Pathological analysis of renal biopsy electron microscopy revealed thin basement membrane nephropathy, a rare autosomal recessive genetic disorder. Deltas et al. [21] have suggested that individuals with thin basement membrane nephropathy are more susceptible to developing FSGS at a later stage. Therefore, it is recommended to carry out genetic screening for uncommon disorders during routine prenatal examination.

## Conclusion

Patients with stages 1–2 of CKD who show good control of important indicators during pregnancy, can actively seek to conceive, leading to better pregnancy and fetal outcomes. It is recommended to exercise caution during pregnancy and terminate it if necessary for patients with pregnancy-related renal impairment with poor clinical control,in order to prevent adverse pregnancy outcomes. Renal biopsy serves as the primary diagnostic basis for determining the cause of kidney damage. The findings of this study suggest that FSGS mainly causes pregnancy-related renal damage, resulting in poor pregnancy outcomes and a high incidence of Scr abnormalities during pregnancy. Therefore, comprehensive examinations in the early stages of pregnancy need to be strengthened, in order to detect kidney disease and intervene early. Currently, there are fewer reports on pregnancy and renal pathology biopsy. More clinical cases require further research from multiple centers.

## Data Availability

The datasets are not publicly available due to the hospital policy and personal privacy, but are available from the corresponding author on reasonable request.
